# FEASIBILITY, ACCEPTABILITY, AND UTILITY OF THE HOME ALONE INTERVENTION: A PRELIMINARY MIXED METHODS EVALUATION

**DOI:** 10.1093/geroni/igae098.4193

**Published:** 2024-12-31

**Authors:** Grace Savard, Robyn Birkeland, Stephanie Ingvalson, Renèe Pepin, Jill Cigliana, Laura Gitlin, Joseph Gaugler

**Affiliations:** University of Minnesota; University of Minnesota, Minneapolis, Minnesota, United States; University of Minnesota, Minneapolis, Minnesota, United States; Geisel School of Medicine at Dartmouth, Lebanon, New Hampshire, United States; Memory Care Home Solutions, Memory Care Home Solutions, Missouri, United States; Drexel University, Philadelphia, Pennsylvania, United States; University of Minnesota, Minneapolis, Minnesota, United States

## Abstract

Research has established that living alone with cognitive impairment in later life presents an increased risk for social isolation, decline in mobility, and other adverse health outcomes. While the number of older adults living independently with cognitive impairment is expected to increase, few programs are available to address the specific needs of this growing population. Home Alone—a novel intervention that includes seven coaching sessions focused on home safety, lifestyle planning, and meaningful engagement through behavioral activation—was designed to support older adults living alone at home with memory concerns. A feasibility pilot phase of Home Alone was conducted between June 2023 and February 2024. Fifteen participants (average age: 73; range: 55-88) living across the United States completed the intervention via telehealth or local in-home sessions. Data and feedback were provided through surveys (baseline, 1, and 3 months) and semi-structured interviews. Mixed methods results showed that the intervention was feasible, acceptable, and useful to participants. Interviews, open-ended survey responses, and coaching sessions yielded insights into program effectiveness and benefits. While in the program, participants restructured home environments to enhance safety, connected with previously unfamiliar resources, and incorporated technology to assist with tasks of daily living. Nearly all participants noted appreciation for tailored activities, educational information, and personal companionship as they navigated transitions and changes. Incorporating participant and staff perspectives from the feasibility phase, this poster will expand on the Home Alone program’s feasibility, acceptability, and utility and highlight modifications made to the intervention for the ongoing larger pilot study (April 2024-Present; n=20).

